# Supplementary material for: TANGO2 binds crystallin alpha B and its loss causes desminopathy
Source: Nat Commun. 2025 Jun 6;16:5261. doi: 10.1038/s41467-025-60563-1 (PMC12144310; doi:10.1038/s41467-025-60563-1)
Supplement: Supplementary file 1 — Supplementary Information [file 41467_2025_60563_MOESM1_ESM.pdf]

Supplementary Information

TANGO2 binds crystallin alpha B and its loss causes desminopathy

Maike Stentenbach[1,2], Laetitia A. Hughes[1,2], Samuel V. Fagan [1,2], Blake Payne[1,2], Danielle L. Rudler[1,2], Stefan J. Siira[1,2], Tim McCubbin[3,4], Anaëlle Chopin[1,2], Kara L. Perks[1], Judith A. Ermer[2], James Hendry[1,2], Teagan S. Er[5], Shanti Balasubramaniam[6,7], Joel A. Eliades[8], Livia C. Hool[5,9], Nicolle H. Packer[10,11], Edward S.X. Moh[10,11], Benjamin S. Padman[1,2], Oliver Rackham[1,2,4,12,13*] and Aleksandra Filipovska[1,2,8,13*]

[1]The Kids Research Institute Australia, Northern Entrance, Perth Children′s Hospital, 15 Hospital Avenue, Nedlands, Western Australia, 6009, Australia

[2]ARC Centre of Excellence in Synthetic Biology, University of Western Australia, Crawley, Western Australia, 6008, Australia

[3]Australian Institute for Bioengineering and Nanotechnology, The University of Queensland, Queensland, 4072, Australia

[4]ARC Centre of Excellence in Synthetic Biology, The University of Queensland, Queensland, 4072, Australia

[5]School of Human Sciences, The University of Western Australia, Crawley, WA, 6009 Australia.

[6]Genetic Metabolic Disorders Service, The Children’s Hospital at Westmead, Sydney, NSW 2145, Australia

[7]Discipline of Genomic Medicine, Sydney Medical School, University of Sydney, Sydney, NSW 2006, Australia

[8]Department of Biochemistry and Molecular Biology, Monash Biomedicine Discovery Institute, Monash University, Melbourne, Australia

[9]Victor Chang Cardiac Research Institute, Darlinghurst, NSW, Australia.

[10]ARC Centre of Excellence in Synthetic Biology, Macquarie University, Sydney, New South Wales, 2109, Australia.

[11]School of Natural Sciences, Macquarie University, Sydney, New South Wales, 2109, Australia.

[12]Curtin Medical School, Curtin University, Bentley, Western Australia, 6102, Australia

Supplementary Information

[13]Curtin Medical Research Institute, Curtin University, Bentley, Western Australia, 6102, Australia

Research Institute, Curtin University, Bentley, Western Australia, 6102, Australia

*Lead Contact for correspondence: oliver.rackham@curtin.edu.au or aleksandra.filipovska@uwa.edu.au

Supplementary Information

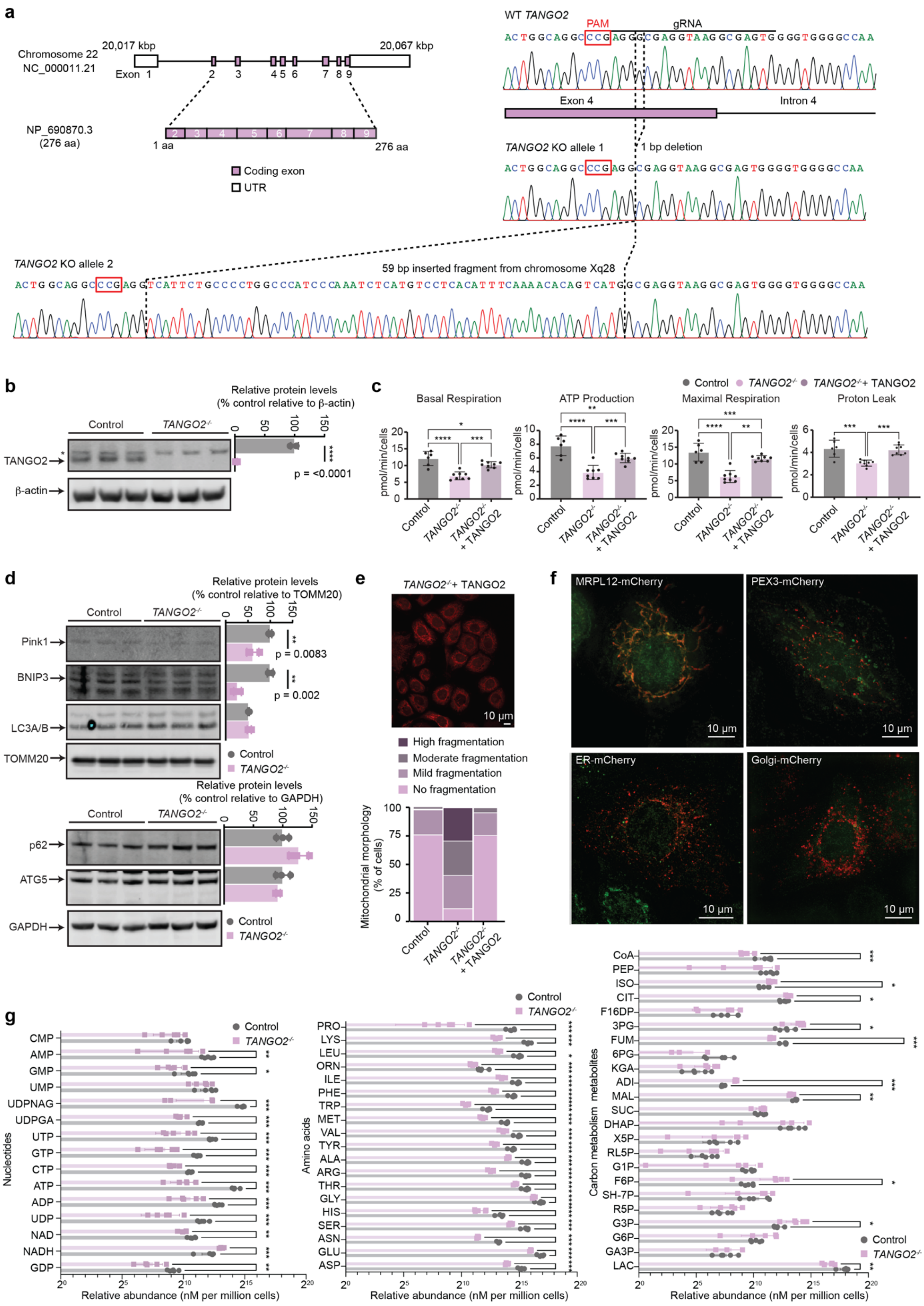

Supplementary Information

Supplementary Figure 1. TANGO2 localization and knockout effects on autophagy and metabolism. (**a**) Confirmation of *TANGO2* disruption in CAL51 cells by Sanger sequencing. (**b**) Immunoblots probed for TANGO2 confirming *TANGO2* knockout. β-actin was used as a loading control (n=3). Values are means ± SD. ****p < 0.0001 Student's two-tailed *t* test. (**c**) Mitochondrial respiration was measured in control, $TANGO2^{-/-}$ and $TANGO2^{-/-}$ rescued with wild-type *TANGO2* cells using a high-resolution respirometer (n=8 per genotype), values are shown as means ± SD *$p < 0.05$, **$p < 0.01$ ***$p < 0.001$, ****$p < 0.0001$, Student's two tailed *t* test. (**d**) Mitochondrial proteins from control and $TANGO2^{-/-}$, cells were resolved by SDS-PAGE and immunoblotted with for PINK1, BNIP3, LC3A/B, p62 and ATG5 (n=3). TOMM20 or GAPDH were used as loading controls. Relative abundance of proteins shown in panels **b** and **d** was analysed relative to the loading control. Values are means ± SD. **$p < 0.01$, ****p < 0.0001 Student's two-tailed *t* test. (**e**) Mitotracker staining of $TANGO2^{-/-}$ cells expressing wild-type TANGO2 grown in glucose media compared to control and $TANGO2^{-/-}$ cells shown in Fig 1i. Cells were incubated with 50 nM Mitotracker Orange prior to fixation and scored for fragmentation (n=100). (**f**) Co-localisation of TANGO2 with other organelles. CAL51 cells with stable expressed TANGO2-GFP were transfected with MRPL12-, PEX3-, ER- or Golgi-mCherry plasmids. Scale bars, 10 μm. (**g**) Metabolomic change in $TANGO2^{-/-}$ cells compared with controls. Relative levels of metabolites are shown as means ± SD *$p < 0.05$, **$p < 0.01$ ***$p < 0.001$, ****$p < 0.0001$, Student's two tailed *t* test (n=5). Source data are provided as a Source Data file.

Supplementary Information

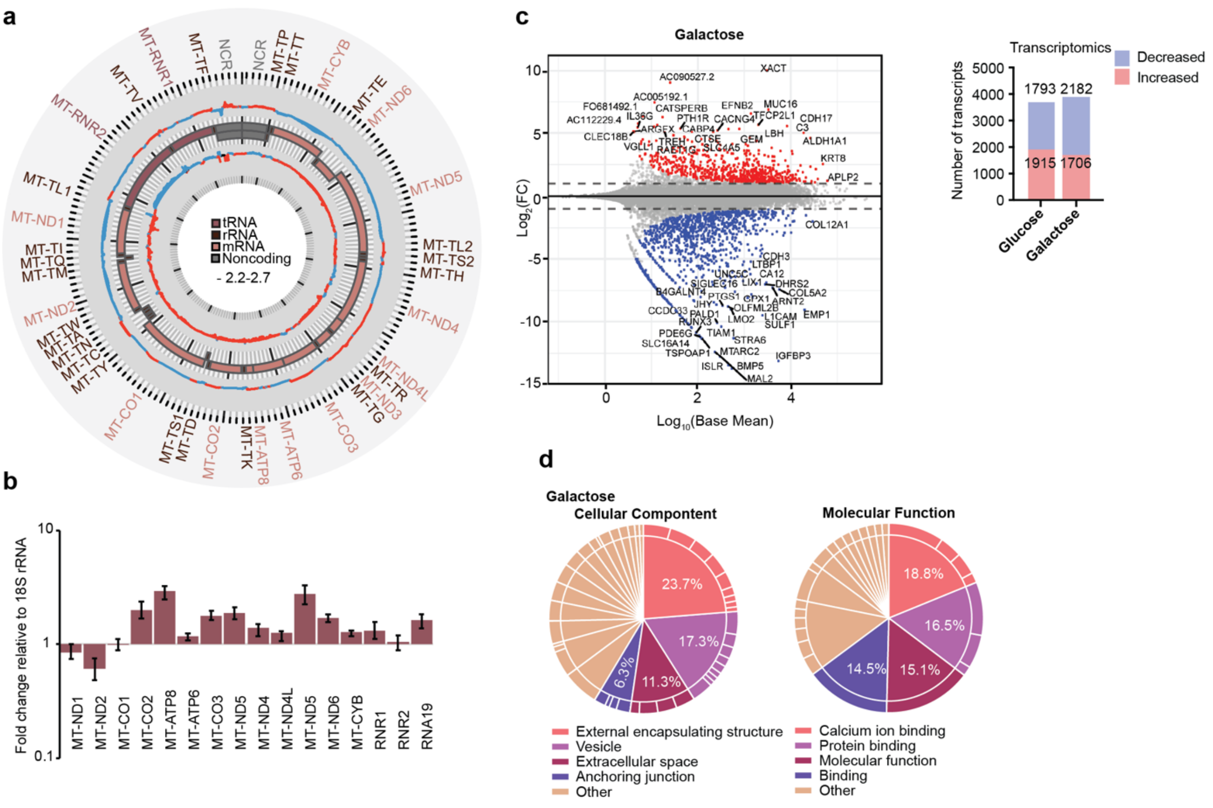

Supplementary Figure 2. Transcriptome-wide changes in *TANGO2* knockout cells grown in galactose. (**a**) Complete map of changes in the mitochondrial transcriptome determined by RNA-Seq coverage from *TANGO2*$^{-/-}$ cells compared with control cells on heavy (outer track) and light (inner track) strands. Increases are shown in red, and decreases are shown in blue (log2[RPMKO/RPMWT]; scale, −2.2 to 2.7). (**b**) Changes in RNA levels analysed by qPCR. RNA levels were normalised to 16S rRNA. (**c**) Transcriptome-wide changes in *TANGO2*$^{-/-}$ cells grown in galactose compared to control cells. Increased genes in red and decreased genes in blue. (**d**) Gene ontology analysis based on transcriptomic changes showing significantly changing pathways involved in molecular function and cellular component.

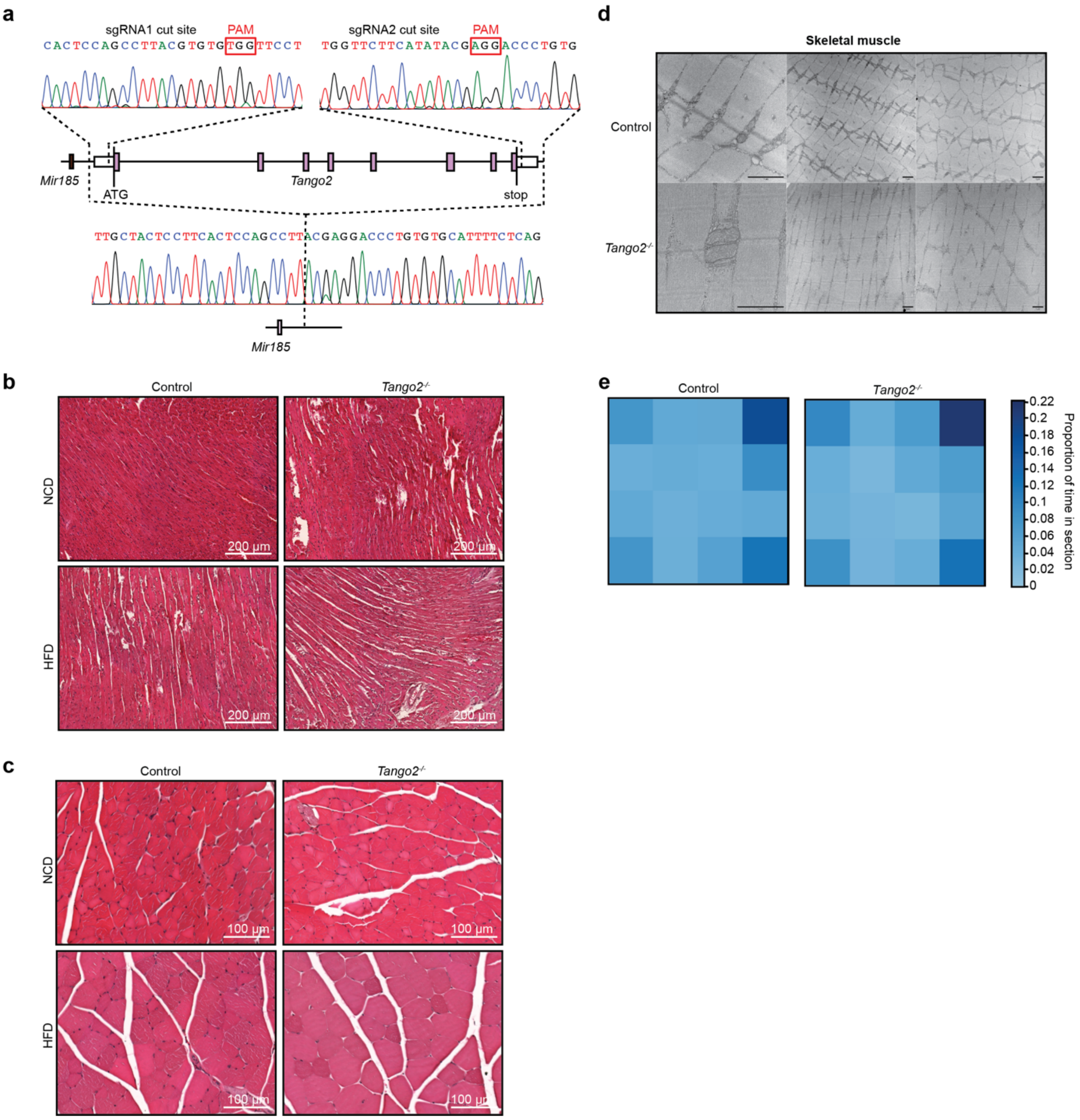

Supplementary Figure 3. Loss of TANGO2 *in vivo* causes structural changes in heart and skeletal muscle. (**a**) Confirmation of *Tango2* deletion in C57BL/6N mice by Sanger sequencing. Hematoxylin and eosin (H&E) staining of 5-µm thick (**b**) heart and (**c**) skeletal muscle sections from 20-week-old control and *Tango2*$^{-/-}$ mice fed a NCD or HFD. Scale bars, 100 µm and 200 µm. (**d**) Electron microscopy images of skeletal muscle sections from 20-week-old from 20-week-old control and *Tango2*$^{-/-}$ mice fed a NCD, scale bar is 1 µm. (**e**) An open field experiment where movement and time spent in each square was measured over 10 minutes (n = 7-10).

Supplementary Information

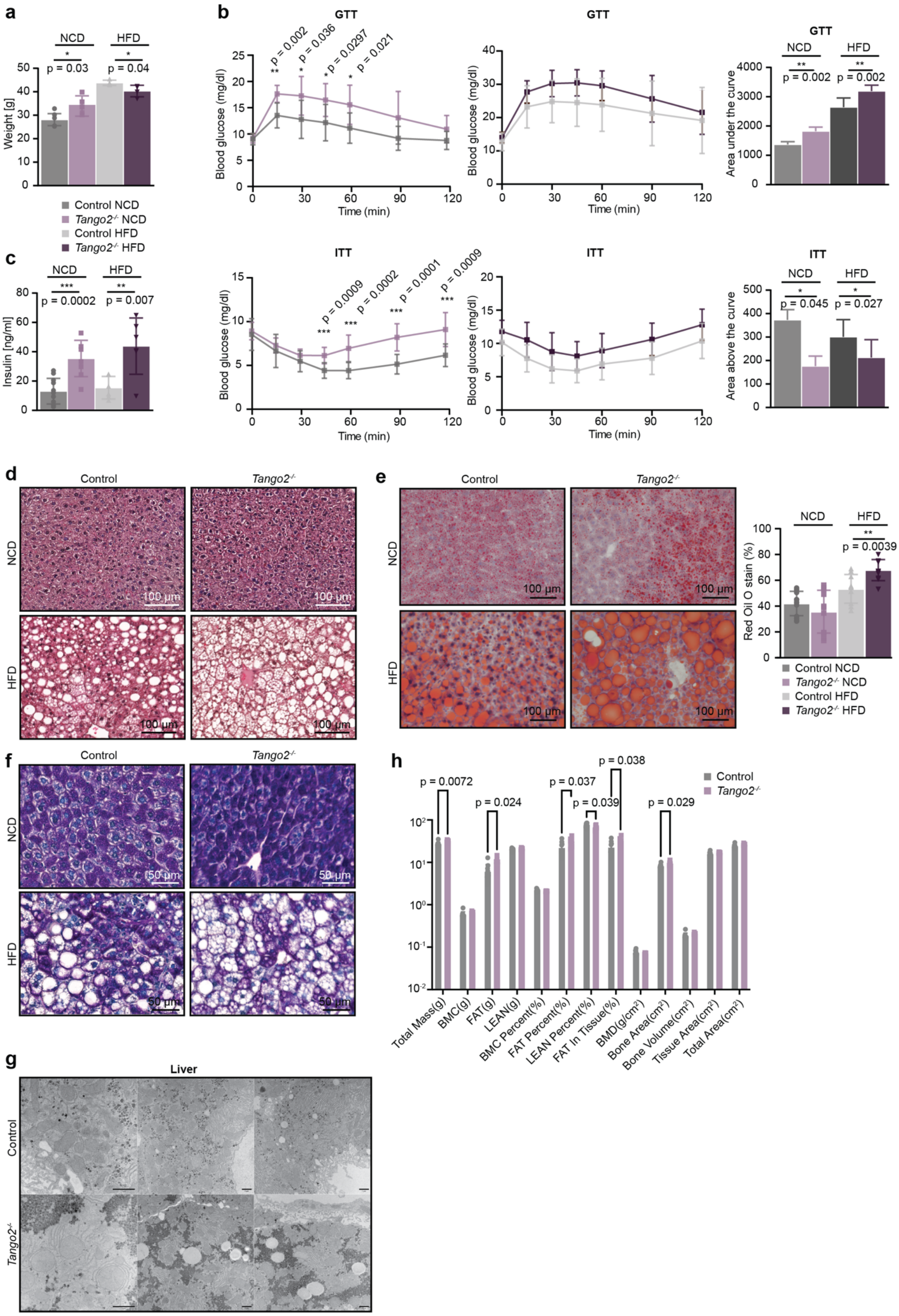

Supplementary Figure 4. *Tango2* deletion leads to insulin resistance and liver steatosis in vivo. (**a**) Body weight of NCD- and HFD-fed control and *Tango2*$^{-/-}$ mice measured at 20 weeks of age (n= >5). (**b**) Glucose tolerance tests (GTT) and insulin tolerance tests (ITT) were carried out in mice fed NCD or HFD at the age of 18 and 19 weeks. Quantitative values are area above the curve for GTT and area under the curve for ITT (n= >5). (**c**) Circulating insulin levels were measured in serum obtained from fasted 20-week-old control and *Tango2*$^{-/-}$ (n = >6) mice fed either a NCD or HFD. All values shown in **a-c** are means ± SD *$p < 0.05$, **$p < 0.01$ ***$p < 0.001$ Student's two-tailed *t* test. (**d**) Hematoxylin and eosin (H&E) staining, (**e**) Oil red O stain and (**f**) periodic acid-Schiff (PAS) stain of 5-μm thick liver sections from 20-week-old control and*Tango2*$^{-/-}$ mice fed a NCD or HFD. Values are means ± SD *$p < 0.05$ Student's two-tailed *t* test. Positive oil red O and PAS stain were quantified using Nikon software. Scale bars, 100 μm. (**g**) Electron microscopy images of livers from 20-week-old control and *Tango2*$^{-/-}$ mice fed a NCD, scale bar is 1 μm. Data in **d-g** are representative of at least 5 biologically independent mice of each genotype. (**h**) Dual energy x-ray absorptiometry was used to analyse the body composition of 20-week-old control and *Tango2*$^{-/-}$ mice fed a NCD. All values are means ± SD *$p < 0.05$, **$p < 0.01$ ***$p < 0.001$ Student's two-tailed *t* test. Source data are provided as a Source Data file.

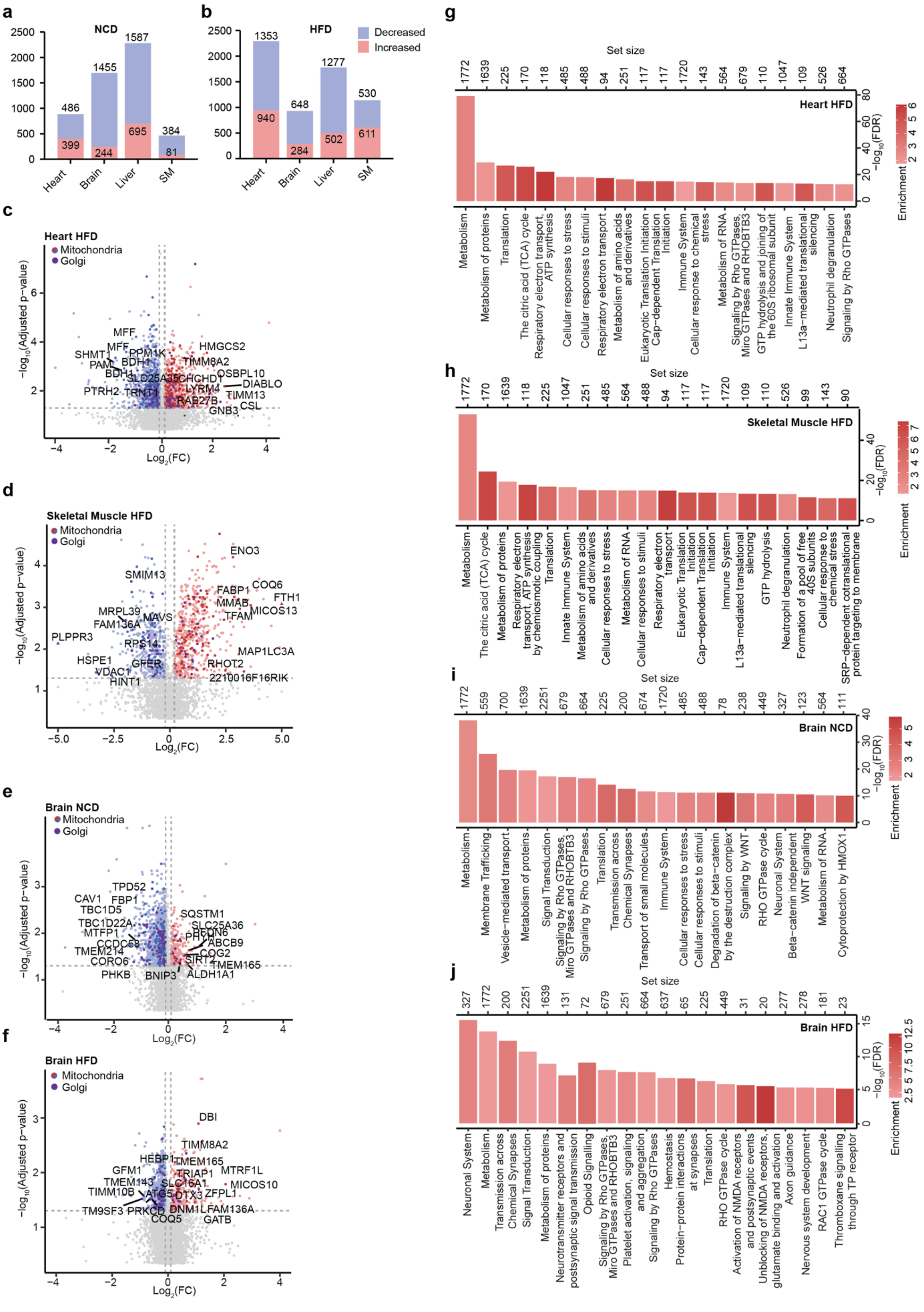

Supplementary Figure 5. Loss of TANGO2 in heart and skeletal muscle affects protein glycosylation and pathways involved in energy metabolism, protein synthesis and

transport. Overall protein changes in heart, liver, skeletal muscle and brain homogenates from 20-week-old *Tango2*$^{-/-}$ mice fed on (**a**) NCD or (**b**) HFD compared with control mice. Increased proteins are shown in red, and decreased proteins are shown in blue. Proteomic changes in (**c**) hearts and (**d**) skeletal muscle from 20-week-old *Tango2*$^{-/-}$ mice fed a HFD compared to control mice (n=5) and brains from 20-week-old *Tango2*$^{-/-}$ mice fed a NCD (**e**) or (**f**) HFD compared to control mice (n=5). Significantly increased and decreased proteins were shown in light red and light blue, respectively, mitochondrial proteins that are increased are in dark red and decreased in dark blue, Golgi proteins are shown in purple. Gene ontology analyses show significantly changing reactome pathways in (**g**) hearts and (**h**) skeletal muscle from 20-week-old *Tango2*$^{-/-}$ mice fed a HFD compared to control mice (n=5) and in brains from 20-week-old *Tango2*$^{-/-}$ mice fed a NCD (**i**) or (**j**) HFD compared to control mice (n=5). The results show the top 20 pathways with the highest $–\log_{10}$(FDR) and the colour scale represents fold change (FC) for each pathway and set size is the number of genes within each pathway.

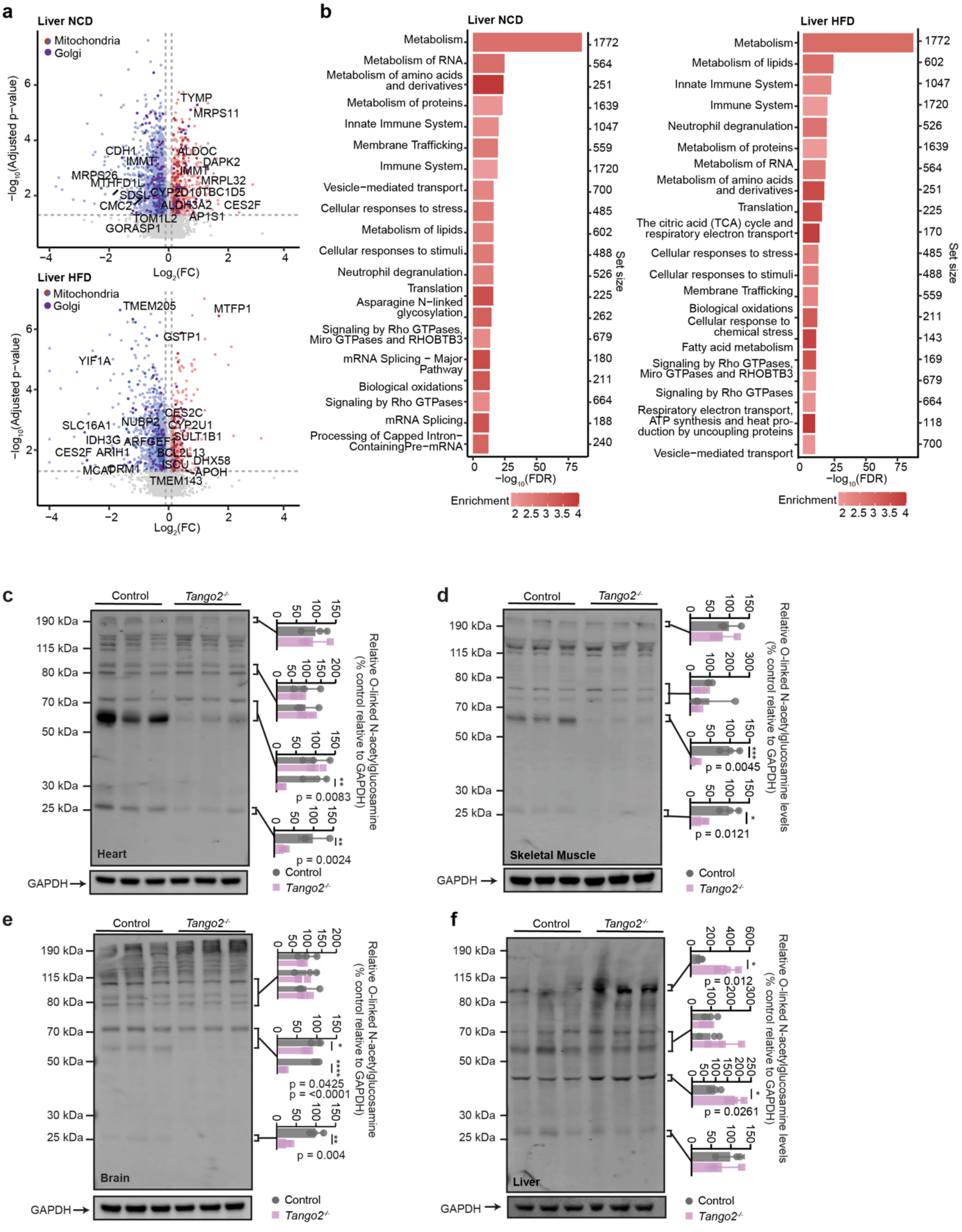

Supplementary Figure 6. The effects of TANGO2 loss in brain and liver on the total proteomes and protein glycosylation. (**a**) Proteomic changes in livers from 20-week-old *Tango2*$^{-/-}$ mice fed a NCD or a HFD compared to their respective control mice (n=5). Significantly increased and decreased proteins were shown in light red and light blue,

respectively, mitochondrial proteins that are increased are in dark red and decreased in dark blue, Golgi proteins are shown in purple. (**b**) Gene ontology analyses show significantly changing reactome pathways in livers from 20-week-old *Tango2*$^{-/-}$ mice fed a NCD or a HFD compared to their respective control mice (n=5). The results show the top 20 pathways with the highest $-\log_{10}$(FDR) and the colour scale represents fold change (FC) for each pathway and set size is the number of genes within each pathway. Immunoblots probed with O-GlcNAc in (**c**) hearts, (**d**) skeletal muscle, (**e**) brains and (**f**) livers from 20-week-old *Tango2*$^{-/-}$ and control mice fed a NCD. GAPDH was used as a loading control. All values in **c-f** are means ± SD *$p < 0.05$, **$p < 0.01$ **$p < 0.001$, ****$p < 0.0001$ Student's two-tailed *t* test. Source data are provided as a Source Data file.

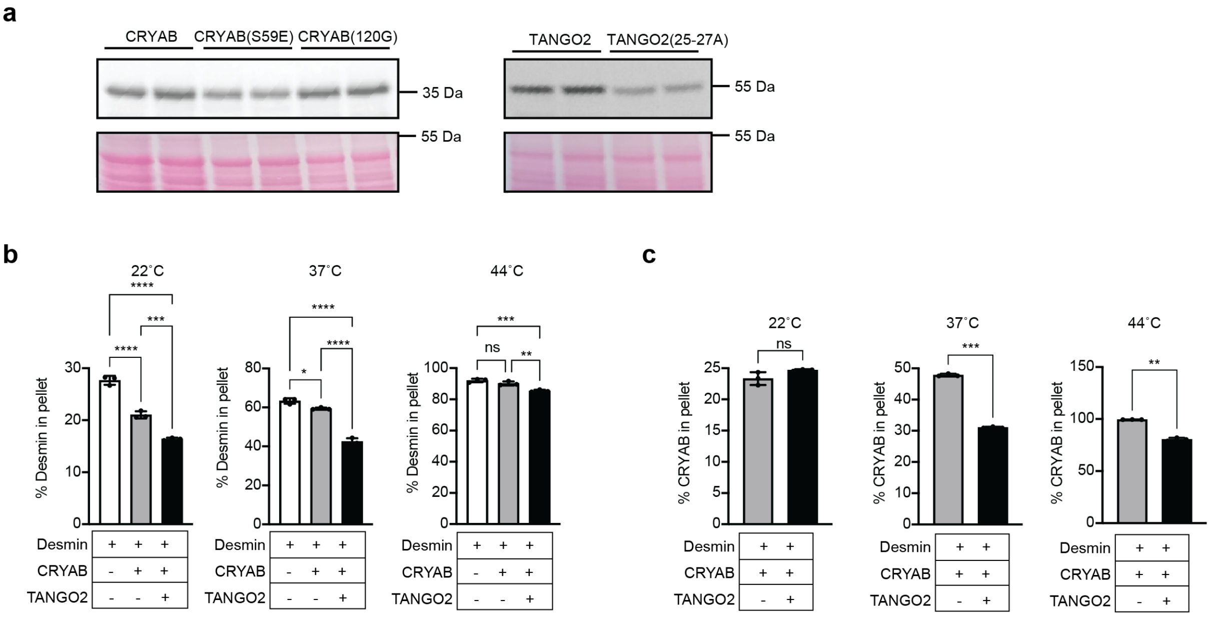

Supplementary Figure 7. (**a**) Immunodetection of wild-type and mutant TANGO2 and CRYAB protein fusions used in yeast two-hybrid assays (n = 4, biological replicates). Ponceau S-stained membranes are shown as loading controls. Quantitation of in vitro assembly of desmin (**b**) and CRYAB (**c**) in the presence or absence of TANGO2. Filament assembly was initiated at 22˚C, 37˚C and 44˚C, and the pellet (P) or aggregated fractions and supernatant (S) or soluble fractions were analysed by SDS-PAGE and stained with Coomassie brilliant blue and quantified using ImageJ. Graphs show the percent levels of desmin (**b**) or CRYAB (**c**) in the pellet. All values are means ± SD *$p < 0.05$, **$p < 0.01$ **$p < 0.001$, ****$p < 0.0001$ Ordinary one-way ANOVA for (**b**) or paired two-tailed *t* test for (**c**). Source data are provided as a Source Data file.

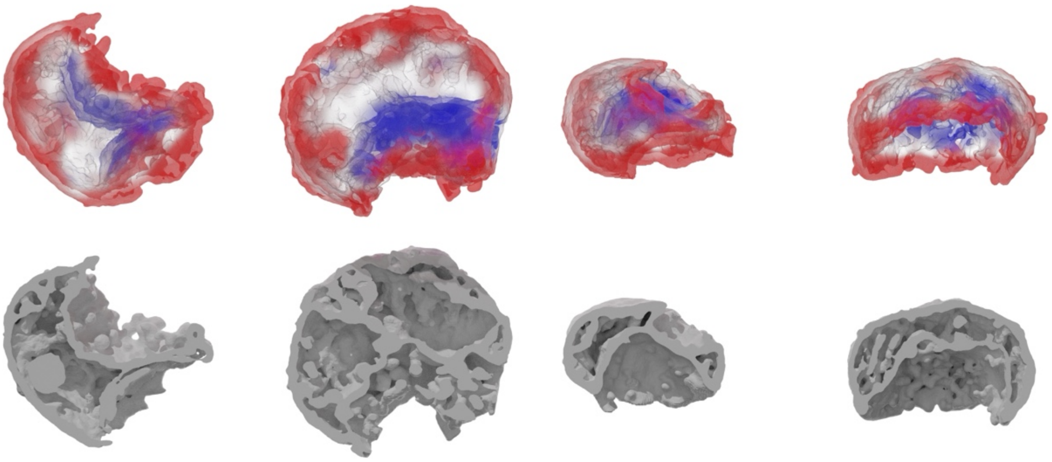

Supplementary Figure 8. Examples of cup-like mitochondria.
