# Supplementary material for: TANGO2 binds crystallin alpha B and its loss causes desminopathy
Source: Nat Commun. 2025 Jun 6;16:5261. doi: 10.1038/s41467-025-60563-1 (PMC12144310; doi:10.1038/s41467-025-60563-1)
Supplement: Supplementary file 2 — Description Of Additional Supplementary File [file 41467_2025_60563_MOESM2_ESM.pdf]

Description of Additional Supplementary Files

Supplementary Data 1. Transcriptome profiles of TANGO2-/- cells grown in glucose or galactose media.

Supplementary Data 2. Proteome profiles of TANGO2-/- cells.

Supplementary Data 3. Glycomic profiles of TANGO2-/- cells.

Supplementary Data 4. In vivo proteome profiles of heart, brain, liver and skeletal muscle in Tango2-/- mice compared to control mice fed a NCD or HFD.

Supplementary Data 5. Yeast-two hybrid screen sequences.

Supplementary Movie 1. Increased filopodia formation in the absence of TANGO2.
