# Supplementary material for: TANGO2 binds crystallin alpha B and its loss causes desminopathy
Source: Nat Commun. 2025 Jun 6;16:5261. doi: 10.1038/s41467-025-60563-1 (PMC12144310; doi:10.1038/s41467-025-60563-1)
Supplement: Supplementary file 9 — Reporting Summary [file 41467_2025_60563_MOESM9_ESM.pdf]

nature portfolio

Corresponding author(s): Aleksandra Filipovska

Last updated by author(s): 30/09/2024

Reporting Summary

Nature Portfolio wishes to improve the reproducibility of the work that we publish. This form provides structure for consistency and transparency in reporting. For further information on Nature Portfolio policies, see our Editorial Policies and the Editorial Policy Checklist.

Statistics

For all statistical analyses, confirm that the following items are present in the figure legend, table legend, main text, or Methods section.

n/a	Confirmed	
☐	☒	The exact sample size (*n*) for each experimental group/condition, given as a discrete number and unit of measurement
☐	☒	A statement on whether measurements were taken from distinct samples or whether the same sample was measured repeatedly
☐	☒	The statistical test(s) used AND whether they are one- or two-sided<br>*Only common tests should be described solely by name; describe more complex techniques in the Methods section.*
☒	☐	A description of all covariates tested
☐	☒	A description of any assumptions or corrections, such as tests of normality and adjustment for multiple comparisons
☐	☒	A full description of the statistical parameters including central tendency (e.g. means) or other basic estimates (e.g. regression coefficient) AND variation (e.g. standard deviation) or associated estimates of uncertainty (e.g. confidence intervals)
☐	☒	For null hypothesis testing, the test statistic (e.g. *F*, *t*, *r*) with confidence intervals, effect sizes, degrees of freedom and *P* value noted<br>*Give P values as exact values whenever suitable.*
☒	☐	For Bayesian analysis, information on the choice of priors and Markov chain Monte Carlo settings
☒	☐	For hierarchical and complex designs, identification of the appropriate level for tests and full reporting of outcomes
☒	☐	Estimates of effect sizes (e.g. Cohen's *d*, Pearson's *r*), indicating how they were calculated

Our web collection on statistics for biologists contains articles on many of the points above.

Software and code

Policy information about availability of computer code

Data collection	No specialised software was used for data collection.
Data analysis	We used the following programs for data analysis, all of which are cited and described in the methods: TrimGalore (v0.6.6), cutadapt31 (v1.18), fastqc (v0.11.9), STAR 2.7, Salmon 1.3.0 , Spectronaut version 16 (16.3.221108.53000) and Blender V2.8.

For manuscripts utilizing custom algorithms or software that are central to the research but not yet described in published literature, software must be made available to editors and reviewers. We strongly encourage code deposition in a community repository (e.g. GitHub). See the Nature Portfolio guidelines for submitting code & software for further information.

Data

Policy information about availability of data

All manuscripts must include a data availability statement. This statement should provide the following information, where applicable:

- Accession codes, unique identifiers, or web links for publicly available datasets
- A description of any restrictions on data availability
- For clinical datasets or third party data, please ensure that the statement adheres to our policy

The authors declare that the data supporting the findings of this study are available within the paper and its extended information files. Genomic data are deposited to the Gene Expression Omnibus (GEO) as GSE276499. The mass spectrometry proteomics data have been deposited to the ProteomeXchange (http://proteomecentral.proteomexchange.org) via the PRIDE partner repository Project accession: PXD056037 and PXD055705 (for BioID experiments). N- and O-glycome raw data files are available via GlycoPost 61 under identifier GPST000473.

Human research participants

Policy information about studies involving human research participants and Sex and Gender in Research.

Reporting on sex and gender	Not applicable
Population characteristics	Not applicable
Recruitment	Not applicable
Ethics oversight	Not applicable

Note that full information on the approval of the study protocol must also be provided in the manuscript.

Field-specific reporting

Please select the one below that is the best fit for your research. If you are not sure, read the appropriate sections before making your selection.

☒ Life sciences ☐ Behavioural & social sciences ☐ Ecological, evolutionary & environmental sciences

For a reference copy of the document with all sections, see nature.com/documents/nr-reporting-summary-flat.pdf

Life sciences study design

All studies must disclose on these points even when the disclosure is negative.

Sample size	No statistical methods were used to predetermine sample size. The minimal sample size was n=3.
Data exclusions	No data were excluded.
Replication	All experiments were performed with a minimum of three independent replicates and replication was successful.
Randomization	No randomization was performed in this study, as no experimental groups were allocated.
Blinding	No blinding was performed in this study, as no group allocation was conducted.

Reporting for specific materials, systems and methods

We require information from authors about some types of materials, experimental systems and methods used in many studies. Here, indicate whether each material, system or method listed is relevant to your study. If you are not sure if a list item applies to your research, read the appropriate section before selecting a response.

Materials & experimental systems

n/a	Involved in the study
[ ]	[x] Antibodies
[ ]	[x] Eukaryotic cell lines
[x]	[ ] Palaeontology and archaeology
[ ]	[x] Animals and other organisms
[x]	[ ] Clinical data
[x]	[ ] Dual use research of concern

Methods

n/a	Involved in the study
[x]	[ ] ChIP-seq
[ ]	[x] Flow cytometry
[x]	[ ] MRI-based neuroimaging

Antibodies

Antibodies used	Specific proteins were detected using rabbit antibodies against LRP130 (sc66844, Santa Cruz Biotechnology; diluted 1:1000), MRPL44 (16394-1-AP, Proteintech, diluted 1:500), MRPS34 (HPA042112-100, Sigma, diluted 1:500), MRPS16 (16735-1-AP, Proteintech, diluted 1:1000), TFAM (HPA040648, Sigma, diluted 1:500), HSD17B10 (HPA001432, Sigma, diluted 1:500), HSP60 (ab137706, Abcam, diluted 1:1000), MTCO2 (ab198286, Abcam, diluted 1:1000), GAPDH (2118s, Cell signaling, diluted 1:1000), NDUFAB (PA5-22191, Thermo Scientific, diluted 1:500), PINK1 (ab23707, Abcam, diluted 1:500), OXA1-L (21055-1-AP, Proteintech, diluted 1:1000), CPT II (0AAN00972, Aviva, diluted 1:1000), LONP1 (ab103909, Abcam, diluted 1:500), AFG3L2 (14631-1-AP, Proteintech, diluted 1:500), CLPX (HPA040262, Sigma, diluted 1:200), LC3A/B (12741, Cell Signaling, diluted 1:500), YME1L1 (ab170123, Abcam, diluted 1:1000), CHCHD3 (ARP57040, Aviva, diluted 1:500), ATG5 (ab108327, Abcam, diluted 1:1000), YY1 (ab109228, Abcam, diluted 1:500), CRYAB (15808-1-AP, Proteintech, diluted 1:500), Vimentin (3932, Cell signaling, diluted 1:1000), SH3GL2 (PA5-120939, Invitrogen, diluted 1:500) and TOMM20 (MA5-32148, Invitrogen, diluted 1:1000) and mouse antibodies against p62 (ab56416, Abcam, diluted 1:500), OPA1 (ab119685, Abcam, diluted 1:500) ATP5a (ab14748, Abcam, diluted 1:500), UQCRC2 (ab14745, Abcam, diluted 1:500), COX IV (ab14744, Abcam, diluted 1:500), BNIP3 (ab10433, Abcam, 1:1000) SDHA (ab14175, Abcam, diluted 1:1000), NDUFA9 (ab14713, Abcam, diluted 1:1000), GFAP (3670, Cell signaling, diluted 1:1000), Total OXPHOS Antibody Cocktail (ab110413, Abcam, diluted 1:1000), VDAC1/Porin (ab14734, Abcam, diluted 1:1000), Desmin (67793-1-Ig, Proteintech, diluted 1:1000) and O-GlcNAc (MA1-072, Invitrogen, 1:1000) in Odyssey Blocking Buffer (Li-Cor). IR Dye 800CW Goat Anti-Rabbit IgG or IRDye 680LT Goat Anti-Mouse IgG (Li-Cor) secondary antibodies were used, and the immunoblots were visualized using the Odyssey Infrared Imaging System (Li-Cor).
Validation	Validation was done by the commercial companies from which the antibodies were purchased.

Eukaryotic cell lines

Policy information about cell lines and Sex and Gender in Research

Cell line source(s)	CAL51 cells (DSMZ, ACC-302)
Authentication	STR profiling.
Mycoplasma contamination	Tested and confirmed negative for mycoplasma contamination.
Commonly misidentified lines (See ICLAC register)	No commonly misidentified cell lines were used in this study.

Animals and other research organisms

Policy information about studies involving animals; ARRIVE guidelines recommended for reporting animal research, and Sex and Gender in Research

Laboratory animals	We used male mice (Mus musculus) on a C57BL/6N background strain that were 10 and 20 weeks old.
Wild animals	The study did not involve wild animals.
Reporting on sex	Only male mice were used in the study and they were identified by visual inspection.
Field-collected samples	The study did not involve samples collected from the field.
Ethics oversight	The Animal Ethics Committee of The University of Western Australia approved the work.

Note that full information on the approval of the study protocol must also be provided in the manuscript.

Flow Cytometry

Plots

Confirm that:

- [x] The axis labels state the marker and fluorochrome used (e.g. CD4-FITC).
- [x] The axis scales are clearly visible. Include numbers along axes only for bottom left plot of group (a 'group' is an analysis of identical markers).
- [x] All plots are contour plots with outliers or pseudocolor plots.
- [x] A numerical value for number of cells or percentage (with statistics) is provided.

Methodology

Sample preparation	Cal51 cells were trypsinised and resuspended in PBS +2% FBS (v/v) for analysis.
Instrument	BD FACSAria II
Software	FlowJo
Cell population abundance	200,000 cells
Gating strategy	No gating strategy is employed and no statistical data is extracted for these plots.

- [x] Tick this box to confirm that a figure exemplifying the gating strategy is provided in the Supplementary Information.
